# Transcriptomic analysis of the cerebral hippocampal tissue in spontaneously hypertensive rats exposed to acute hypobaric hypoxia: associations with inflammation and energy metabolism

**DOI:** 10.1038/s41598-023-30682-0

**Published:** 2023-03-06

**Authors:** Wei Chang, Jinxiu Cui, Yajuan Li, Kehai Zang, Xutao Zhang, Zhuoru Zhang, Yihong Jiang, Qianqian Ma, Shuai Qu, Fengzhou Liu, Junhui Xue

**Affiliations:** 1 Center for Aerospace Clinical Medicine, Department of Aerospace Medicine, Air Force Medical University, Xi’an, 710032 China; 2 Department of Military Medical Equipment and Metrology, School of Military Biomedical Engineering, Air Force Medical University, Xi’an, 710032 China; 3 The College of Life Sciences, Northwest University, Xi’an 710069, China; 4 Department of Aviation Medicine, Xijing Hospital, Air Force Medical University, Xi’an, 710032 China

**Keywords:** Gene expression, Cardiovascular diseases, RNA sequencing

## Abstract

We evaluated the effect of acute hypobaric hypoxia (AHH) on the hippocampal region of the brain in early-stage spontaneously hypertensive male rats. The rats were classified into a control (ground level; ~ 400 m altitude) group and an AHH experimental group placed in an animal hypobaric chamber at a simulated altitude of 5500 m for 24 h. RNA-Seq analysis of the brains and hippocampi showed that differentially expressed genes (DEGs) were primarily associated with ossification, fibrillar collagen trimer, and platelet-derived growth factor binding. The DEGs were classified into functional categories including general function prediction, translation, ribosomal structure and biogenesis, replication, recombination, and repair. Pathway enrichment analysis revealed that the DEGs were primarily associated with relaxin signaling, PI3K-Akt signaling, and amoebiasis pathways. Protein–protein interaction network analysis indicated that 48 DEGs were involved in both inflammation and energy metabolism. Further, we performed validation experiments to show that nine DEGs were closely associated with inflammation and energy metabolism, of which two (*Vegfa* and *Angpt2*) and seven (*Acta2, Nfkbia, Col1a1, Edn1, Itga1, Ngfr*, and *Sgk1*) genes showed up and downregulated expression, respectively. Collectively, these results indicated that inflammation and energy metabolism-associated gene expression in the hippocampus was altered in early-stage hypertension upon AHH exposure.

## Introduction

Studies have shown the prevalence of cerebrovascular lesions and cognitive impairment in early-stage hypertensive patients and animal models^1–3^. Abnormalities in vascular structure and function, including endothelial dysfunction, increased oxidative stress and vascular remodeling, and decreased compliance are considered symptoms of early-stage hypertension. These phenotypes play an important role in the development of hypertension^4^. Furthermore, varying degrees of brain damage are common under conditions involving acute hypobaric hypoxic (AHH)^5^. Upon first arriving at high altitudes, significant changes are observed in the cerebral hemodynamics of individuals that live at low altitudes. As the degree of hypobaric hypoxia increases, arterial vasodilatation gradually increases, including that in the middle cerebral artery. In turn, pathological changes such as increased intracapillary pressure, loss of autoregulatory function, and abnormal neural and humoral regulation are observed^6^. Inflammation is associated with the susceptibility to and development of high-altitude cerebral edema. Hypoxia enhances lipopolysaccharide-induced inflammation and mediates the onset and development of cerebral edema in mice at high altitudes. This phenotype can be attributed to the disruption of blood–brain barrier integrity and activation of the microglia^7^. Hypobaric hypoxia results in abnormal alterations in the energy metabolism of the body, including changes in various branched-chain amino acids, succinate, lactate, and pyruvate^8–10^. Compared with healthy individuals, those with hypertension exhibit a significantly higher probability of developing acute altitude sickness when entering high-altitude regions^11^. AHH conditions, in addition to hypertension, may lead to an increase in the severity of cerebral damage. However, the underlying pathophysiological mechanisms remain unclear.

Transcriptome analysis is widely used in the study of biological processes (physiological mechanisms, pathways, or genes) associated with cardiovascular and cerebrovascular diseases. RNA-sequencing (RNA-seq) and subsequent bioinformatics analysis have been used to investigate the potential molecular targets associated with energy metabolism under conditions involving hypertension during diabetes. The results have led to the discovery of novel therapeutic targets^12^. A Japanese study performed transcriptomic characterization of samples from individuals under conditions involving AHH. The transcriptional profile of individuals underwent rapid changes under conditions involving acute hypoxic, and these changes may affect individual adaptation to the hypoxic environment^13^.

To investigate the effects of AHH on the hippocampus of early-stage hypertensive brains, we used six-week-old male spontaneously hypertensive rats (SHRs) in our study. SHR rats are the most commonly used animal model to study the physiological mechanism underlying hypertension. The blood pressure of SHR rats began to increase from the fourth to the sixth week^14^. Since sex plays a crucial role in the development of hypertension, and the incidence of early hypertension is significantly higher in men than in women, we considered only male animals in the selection of study subjects^15^. A large number of neural cells exist in the hippocampal region, and their function is closely associated with cognition. Moreover, the hippocampal region has a rich blood supply. The normality of vascular function has a remarkable influence on the role of the hippocampal region. Therefore, we exposed six-week-old SHRs to a hypobaric hypoxic environment at 5500 m or a control ground-level environment (~ 400 m above sea level) for 24 h. Subsequently, we anesthetized the rats and isolated the hippocampal region of the brain for high-throughput RNA-seq. Based on the sequencing results, inflammation-associated genes and energy metabolism-associated genes were screened, and a protein–protein interaction (PPI) network was constructed. Furthermore, the data were validated using quantitative polymerase chain reaction (qPCR). This study aimed to investigate the effects of AHH on the hippocampus in the brain during early-stage hypertension. Furthermore, we aimed to validate the role of inflammation and energy metabolism via transcriptome sequencing and PPI network construction.

## Materials and methods

### Animals and blood pressure measurements

All experiments were performed using six-week-old male SHRs (systolic blood pressure: 151.5 ± 3.5 mmHg; diastolic blood pressure: 109.6 ± 6.0 mmHg) purchased from the Animal Experiment Center of Air Force Medical University, China (Supplementary Table 1). The systolic blood pressure of the caudal artery was measured using a BP-2010A automatic non-invasive blood pressure meter (Softron Biotechnology Co., Ltd, Beijing, China) at 8:00 am in the rats during the resting state. Five measurements were obtained for each rat, and the mean of the five readings was considered as the systolic blood pressure of the rat. All animals (license lot number SCXK 2017–0021) were considered eligible for the experiment. During the experiment, animals were housed at 21 ± 1 °C with ad libitum access to food and water. All animal experiments and operations were performed under the guidance of the Animal Research Committee and approved by the Air Force Medical University Animal Ethics Committee of the Institute (No. IACUC-20220392). All animal feeding and experimental procedures followed the guidelines of the institutional ethics review board of the Air Force Medical University and strictly performed according to the ARRIVE guidelines^16^.

### RNA-seq

Six male SHRs were equally and randomly classified into the ground-level (~ 400 m above sea level) control or the AHH experimental groups. During the experiment, the rats in the control group were fed the same food and water as those in the AHH group. The rats in the AHH group were placed in an animal hypobaric pressure chamber (Department of Aerospace Medicine of Air Force Medical University and Hongyuan Oxygen Industry Co., Ltd., Xi'an, China), elevated to 5500 m at 10–15 m/s, and returned to ground level at a rate of 15–20 m/s after 24 h. The rats in both groups were euthanized with an overdose of 1.5% sodium pentobarbital administered via intraperitoneal injection, and the brains were harvested.

The hippocampus of the brain was isolated, and total RNA was extracted from the tissue using TRIzol (Invitrogen, Carlsbad, CA, USA) according to the manufacturer's instructions. Subsequently, total RNA was quantified and characterized using a Nanodrop spectrophotometer and an Agilent 2100 Bioanalyzer (Thermo Fisher Scientific, Waltham, MA, USA), respectively (28S/18S > 1.0, RIN > 7.0). Hieff NGS® DNA Selection Beads (Yeasen, Shanghai, China) were used for mRNA purification. The purified mRNA was fragmented into small fragments using a fragmentation buffer at an appropriate temperature. First-strand cDNA was generated using random hexamer-initiated reverse transcription, followed by second-strand cDNA synthesis, according to the kit instructions (Takara Bio Inc., Beijing, China). End repair was performed by incubation with an A-tail mixture and an RNA index adapter (Yeasen, Shanghai, China). The cDNA fragment obtained was amplified using qPCR, purified with AMPure XP beads, and eluted with an elution buffer solution. The PCR products were checked for quality using an Agilent Technologies 2100 Bioanalyzer. The double-stranded PCR products obtained were denatured by heating and cyclized with a splint oligo sequence to obtain the final library. Single-stranded circular DNA was used as the final library. DNA nanoballs (DNBs) were amplified using phi29; one molecule had more than 300 copies. The DNBs were loaded into patterned nanoarrays to generate 150 base-pair end reads on the DNBSEQ-T7 platform (Tsingke Biotechnology Co. Ltd., Beijing, China).

### Quality control and identification

The raw reads obtained from the sequencing data were filtered to obtain clean reads and compared to the reference genome of *Rattus norvegicus* mRatBN7.2(7/7/2022). Six samples were sequenced using the Illumina platform, yielding 44.09 GB of data. The percentage of bases with quantitative values higher than 30 (Q30) was ≥ 94.24%. The clean reads of each sample were aligned relative to the designated reference genome separately, and the matching efficiency ranged from 96.66 to 97.27%. The sequencing data were of good quality and met the requirements for subsequent analysis (Table 1). After preprocessing and filtering, gene expression levels were normalized to transcript per kilobase million (TPM) values.

**Table 1:** Statistical analysis of transcriptome sequencing of the hippocampal tissue of spontaneous hypertensive rats (SHRs) exposed to acute hypobaric hypoxia (AHH) for 24 h in the AHH group compared to those in the control group.

**Sample Name**	Clean Reads	Clean Bases	Clean Reads Q30 (%)
Control-24 h-1	21,095,034	6,307,222,116	94.66
Control-24 h-2	22,356,567	6,683,650,606	94.47
Control-24 h-3	31,972,114	9,556,379,680	95.79
AHH-24 h-1	19,693,541	5,885,567,860	95.12
AHH-24 h-2	23,146,556	6,903,908,264	96.50
AHH-24 h-3	29,285,797	8,754,402,342	94.24

Sample name: sample name of the sample information sheet. Clean reads: total number of paired-end reads in the clean data. Clean bases: total number of bases in the clean data number. Clean reads Q30: the percentage of bases with clean data quality value higher than or equal to 30.

### GO and KEGG enrichment analysis of differentially expressed genes (DEGs)

Gene expression was calculated for each sample as TPM values, and the DEseq2 R package (version: 4.1.3) was used for analyzing DEGs with a screening threshold of ∣log_2_(fold change)∣ ≥ 1.2, *p* < 0.05^17^. The functional enrichment analysis of DEG was performed using the clusterProfiler package based on the Gene Ontology (GO) (http://www.geneontology.org) and Kyoto Encyclopedia of Genes and Genomes (KEGG) databases (http://www.genome.jp/kegg/)^17^. The conditional threshold for GO and KEGG analysis was *p* < 0.05.

### Screening of inflammation and energy metabolism-associated genes and PPI network construction

Based on previous studies^18^, genes associated with inflammation were retrieved by searching the GeneCards database (https://www.genecards.org/). A similar search strategy was employed for energy metabolism-associated genes. Based on this, we obtained DEGs associated with both inflammation and energy metabolism. These DEGs were entered into the STRING database (https://string-db.org/) and analyzed, and a PPI network was constructed. The network file was downloaded and imported into Cytoscape (version 3.9.1) and visualized based on the degree of the topological properties of this network^19^.

### Reverse transcription qPCR validation

Total RNA was extracted using TRIzol (DBI Bioscience, Shanghai, China). cDNA was obtained using a reverse transcription kit (DBI Bioscience, Shanghai, China) and amplified using SYBR Green PCR Master Mix (DBI Bioscience, Shanghai, China) according to the manufacturer’s instructions. β-actin was used to normalize gene expression, and the relative gene expression values were calculated using the 2^–∆∆Ct^ method^20^. Primer sequences were retrieved from PrimerBank 40 (Supplementary Table 2), and primer specificity was further validated using the National Center for Biotechnology Information Primer-BLAST tool.

### Statistical analysis of GO, KEGG enrichment, and qPCR results

Based on the GO (http://www.geneontology.org) and KEGG databases (http://www.genome.jp/kegg/), the functions of DEGs can be discerned, and the associated pathways can be enriched using the clusterProfiler package of R. The cutoff of GO enrichment analysis was set to *p* < 0.05. Significant KEGG pathways were screened based on *p* < 0.05. The data of reverse transcription qPCR were statistically analyzed using GraphPad Prism 8.0 software. The data are expressed as the mean ± standard deviation. Comparisons between groups were performed using the independent sample *t*-test, and differences were considered statistically significant at *p* < 0.05, n = 6.

## Results

### Analysis of DEGs

**Figure 1:**
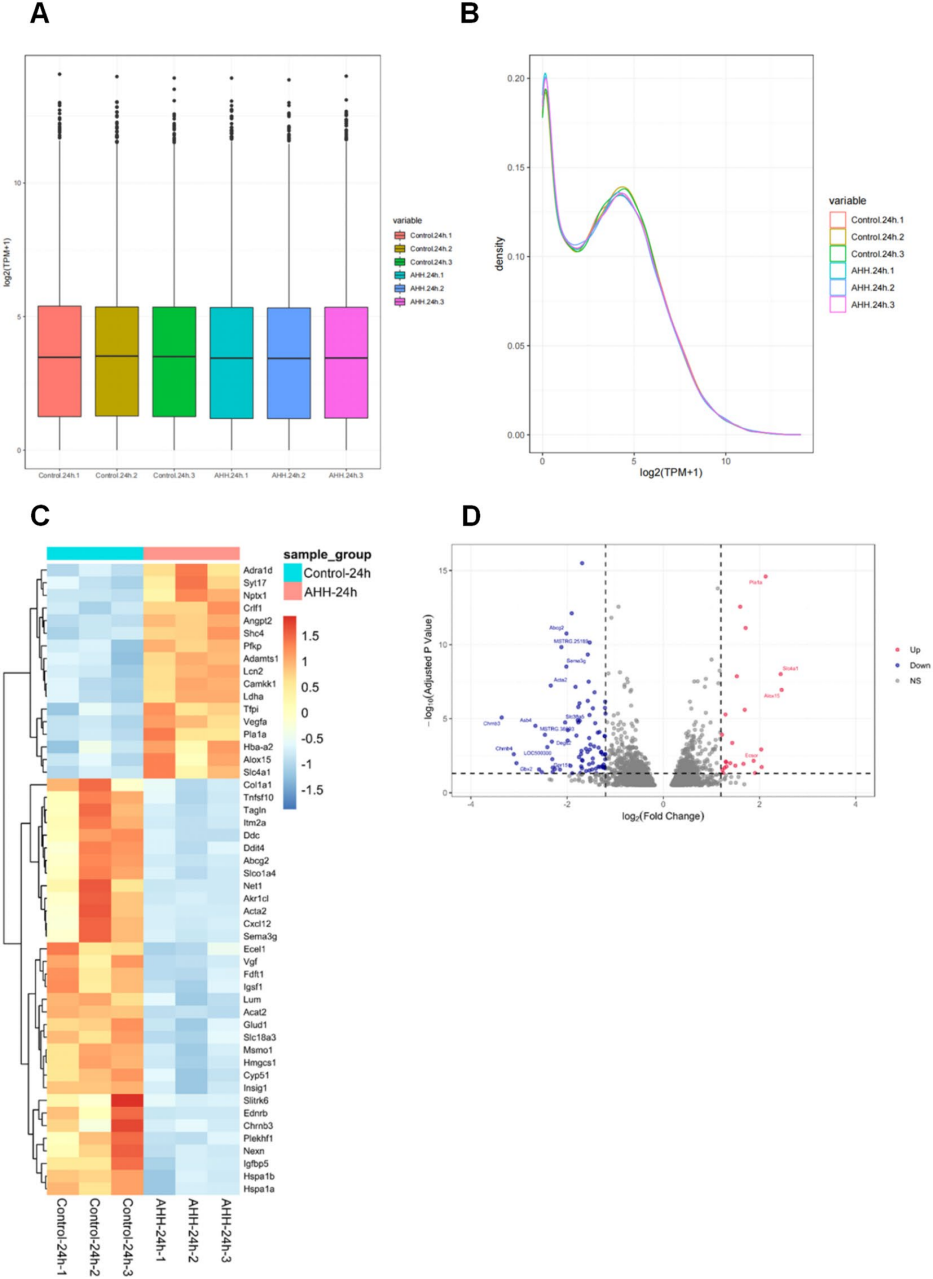
(**A**) Box plot of log_2_ (TPM + 1) expression values for each sample. The horizontal coordinates in the plot represent the different samples; the vertical coordinates indicate the expression level of the sample log_2_ (TPM + 1) values. The plot indicates the expression level of each sample in terms of the overall dispersion of expression. Box line plots for each region correspond to six statistical parameters (upper, outlier, upper quartile, median, lower quartile, and lower limit from top to bottom). (**B**) Density plot of the distribution of log_2_ (TPM + 1) values for each sample. Different colored curves in the graph represent different samples; the horizontal coordinates of the points on the curves indicate the log values of TPM + 1 for the corresponding samples, and the vertical coordinates of the points indicate the probability density. (**C**) Heatmap analysis of 112 differentially expressed genes (DEGs) in the two groups: control-24 h and acute hypobaric hypoxia (AHH)-24 h groups. All genes shown are significantly differentially expressed between the two groups (adjusted *p* < 0.05). Colored bars from blue to red represent the increasing levels of gene expression from low to high. (**D**) Volcano plot analysis of 112 DEGs in the control-24 h and AHH-24 h groups. Each dot represents a gene that was detected in both groups. Red and blue dots indicate genes with significantly up and downregulated expression, respectively. Gray dots show genes that were not significantly differentially expressed between the control-24 h and AHH-24 h groups. The horizontal axis indicates the log ratio (changes in gene expression ploidy across samples), and the vertical axis indicates the probability of differential expression of each gene. The DEGs were identified using a false discovery rate of 0.05.

Boxplots for the distribution of DEGs across all six data sets are shown in Fig. 1A. The density plots show that the two groups (control-24 h and AHH-24 h) each had three replicates with a similar distribution of the six curves (Fig. 1B). In addition, the cluster heatmap shows differences in the gene expression profiles between the AHH and control groups (Fig. 1C). To determine the differential expression of these genes in the hypobaric hypoxic environment, we analyzed changes between the two groups using volcano plots (Fig. 1D). DEGs were filtered based on the criterion ∣log_2_(fold change)∣ ≥ 1.2, *p* < 0.05, and 112 DEGs were subsequently obtained by comparing the hippocampal tissue between the AHH and control groups. Of these 112 genes, 25 and 87 genes showed up- and downregulated expression, respectively, in the AHH group.

### GO-, clusters of orthologous gene (COG)-, and KEGG-based functional classification of DEGs

**Figure 2:**
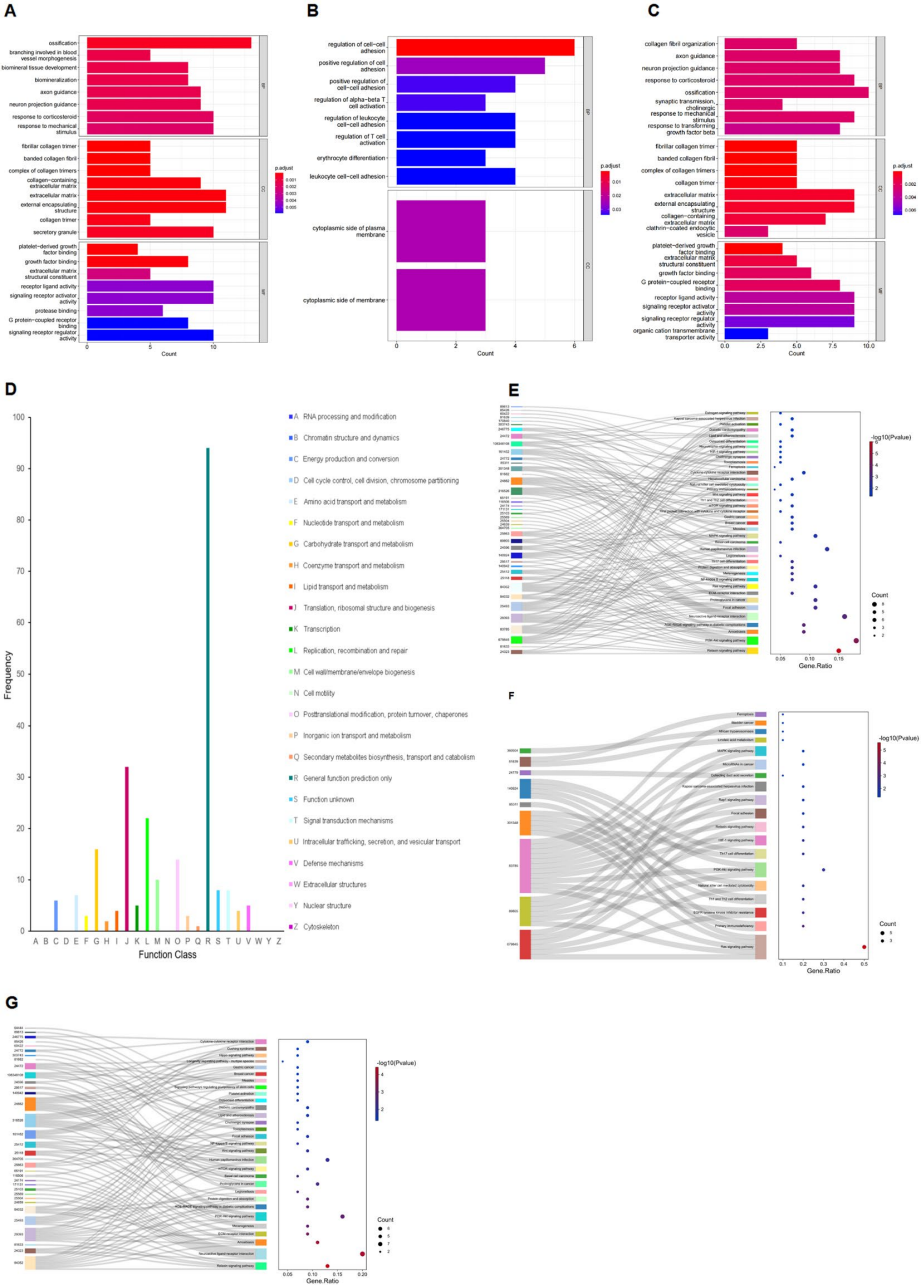
(**A**) Gene ontology (GO) annotation analysis of 112 differentially expressed genes (DEGs) for the control-24 h and acute hypobaric hypoxia (AHH)-24 h groups. The top eight categories with the smallest *p* values for each classification were screened for GO annotations. BP, biological process; CC, cellular component; MF, molecular function. The bar color in the graph represents the *p* value distributed from blue to red; the closer to red, the smaller the *p*-value, and vice versa. (**B**) DEGs upregulated in the AHH-24 h group compared with those in the control-24 h group subjected to GO annotation analysis. (**C**) DEGs downregulated in the AHH-24 h group compared with those in the control-24 h group analyzed by GO annotation. (**D**) Clusters of orthologous gene (COG) classification analysis of 112 DEGs. The vertical axis indicates the frequency of DEGs in specific functional clusters, and the horizontal axis indicates the functional class. (**E**) Kyoto Encyclopedia of Genes and Genomes (KEGG) transcript classification analysis of 112 DEGs in the control-24 h and AHH-24 h groups. The left and right-colored bars represent the two groups of DEGs and the KEGG pathways, respectively. Lines connecting the KEGG pathway show the enrichment of the two sets of DEGs. The horizontal axis of the bubble plot indicates the gene ratio (the ratio of the number of DEGs enriched in the corresponding pathway to the number of all DEGs entered for enrichment analysis). The vertical axis indicates the KEGG pathway term, and the bubble size represents the number of differentially annotated genes in a term, with larger bubbles indicating more genes. The color represents the enrichment significance *p *value, with a higher intensity of the red color representing a smaller value (indicating stronger significance). (**F**) KEGG analysis of DEGs upregulated in the AHH-24 h group compared with those in the control-24 h group. KEGG transcripts for taxonomic analysis. (**G**) KEGG transcript classification analysis of the DEGs downregulated in the AHH-24 h group compared with those in the control-24 h group.

A total of 112 DEGs were annotated using GO analysis and classified into three categories, i.e., biological processes, cellular components, and molecular functions (Fig. 2A and Supplementary Table 3). The upregulated DEGs were primarily enriched in the biological process and cellular component categories. In the biological process module, DEGs were primarily associated with the regulation of cell–cell adhesion, positive regulation of cell adhesion, positive regulation of cell–cell adhesion, regulation of alpha–beta T cell activation, and regulation of leukocyte cell–cell adhesion. In the cellular component module, DEGs with upregulated expression were primarily associated with the cytoplasmic side of the plasma membrane. In addition, DEGs with upregulated expression were not involved in molecular functions (Fig. 2B and Supplementary Table 4). However, DEGs with downregulated expression were highly enriched in several GO functional categories, including collagen fibril organization, axon guidance, and neuron projection guidance in the biological process module, fibrillar collagen trimer, banded collagen fibril; a complex of collagen trimers in the cellular components module; and platelet-derived growth factor binding, extracellular matrix structural constituent, and growth factor binding in the molecular functions module. Interestingly, many subcategories of DEGs showed downregulated expression (Fig. 2C and Supplementary Table 5).

In the COG database, 112 DEGs were classified into 18 functional categories. The main category was general function prediction, followed by translation, ribosomal structure and biogenesis, replication, recombination, and repair, carbohydrate transport and metabolism, posttranslational modification, protein turnover, and chaperones (Fig. 2D and Supplementary Table 6).

KEGG pathway enrichment analysis was performed for both groups of DEGs, and the threshold for determining gene enrichment was *p* < 0.05 (Fig. 2E and Supplementary Table 7). In the KEGG enrichment analysis, the most enriched category was the PI3K-Akt signaling pathway, followed by neuroactive ligand-receptor interaction, relaxin signaling pathway, and human papillomavirus infection. In parallel, analysis of enriched pathways for DEGs with up- and downregulated expression was performed separately. For DEGs with upregulated expression, the most significantly enriched pathways were the Ras and PI3K-Akt signaling pathways (Fig. 2F and Supplementary Table 8). For DEGs with downregulated expression, neuroactive ligand-receptor interaction, PI3K-Akt signaling pathway, human papillomavirus infection, and relaxin signaling pathway were significantly enriched (Fig. 2G and Supplementary Table 9).

### Screening for inflammation and energy metabolism-associated genes

**Figure 3:**
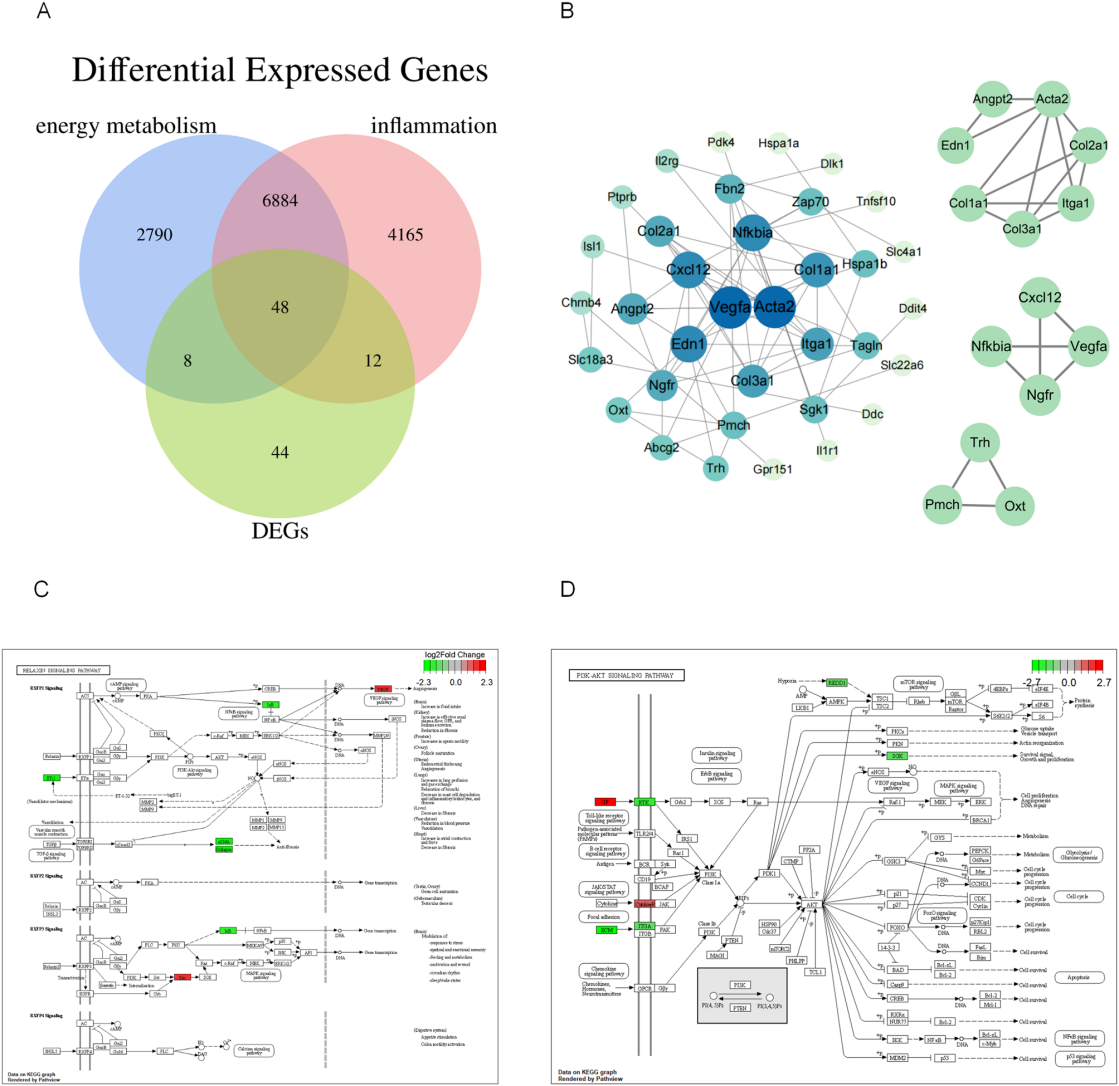
(**A**) Venn diagram of common differentially expressed genes (DEGs) between groups. The three circles represent energy metabolism-associated genes, inflammation-associated genes, and DEGs. The intersecting regions of the circles indicate the intersecting genes of different groups. (**B**) Proteins interacting with inflammation- and energy metabolism-associated genes. The protein–protein interaction network of DEGs was visualized using Cytoscape (version 3.9.1) according to its topological properties. The higher the color intensity and the lower the distance to the center, the more the number of genes it interacted with in the network. The hub sub-networks were screened by the MCODE plug-in. (**C**) Relaxin signaling pathway (www.kegg.jp/entry/map04926). Red and green boxes represent DEGs that were significantly up or downregulated in this pathway, respectively. (**D**) PI3K-Akt signaling pathway (www.kegg.jp/entry/map04151). Red and green boxes represent DEGs that were significantly up or downregulated in this pathway, respectively.

To elucidate the role of DEGs related to inflammation and energy metabolism, the term “energy metabolism” was searched on the GeneCards website to obtain a list of associated genes. Inflammation-associated genes were retrieved in a similar manner. The overlaps between the resultant 9730 energy metabolism-associated genes and 11,109 inflammation-associated genes were analyzed along with the 112 DEGs, yielding 48 overlapping genes (Fig. 3A). We constructed a PPI network using the STRING database to further investigate the biological roles of the DEGs associated with energy metabolism and inflammation (Fig. 3B). Among them, genes encoding vascular endothelial growth factor A (*Vegfa*) and actin alpha 2 (*Acta2*) showed a central pivotal position. Genes encoding nuclear factor of kappa light polypeptide gene enhancer in B-cells inhibitor alpha (*Nfkbia*)*,* collagen type I alpha 1 (*Col1a1*)*,* endothelin 1 (*Edn1*), and serum/glucocorticoid regulated kinase 1 (*Sgk1*) also showed numerous associations with other genes. The ten most significant pathways were screened using KEGG enrichment analysis (*p* < 0.05; Fisher’s exact test, followed by the Bonferroni test). The results showed significant enrichment of the relaxin signaling and PI3K-Akt signaling pathways, among others (Fig. 3C,D, and Supplementary Table 10). The DEGs evaluated via PPI analysis indicate a vital role of signaling pathways related to energy metabolism and inflammation in the response to AHH.

### Validation of RNA-seq data using qPCR

**Figure 4:**
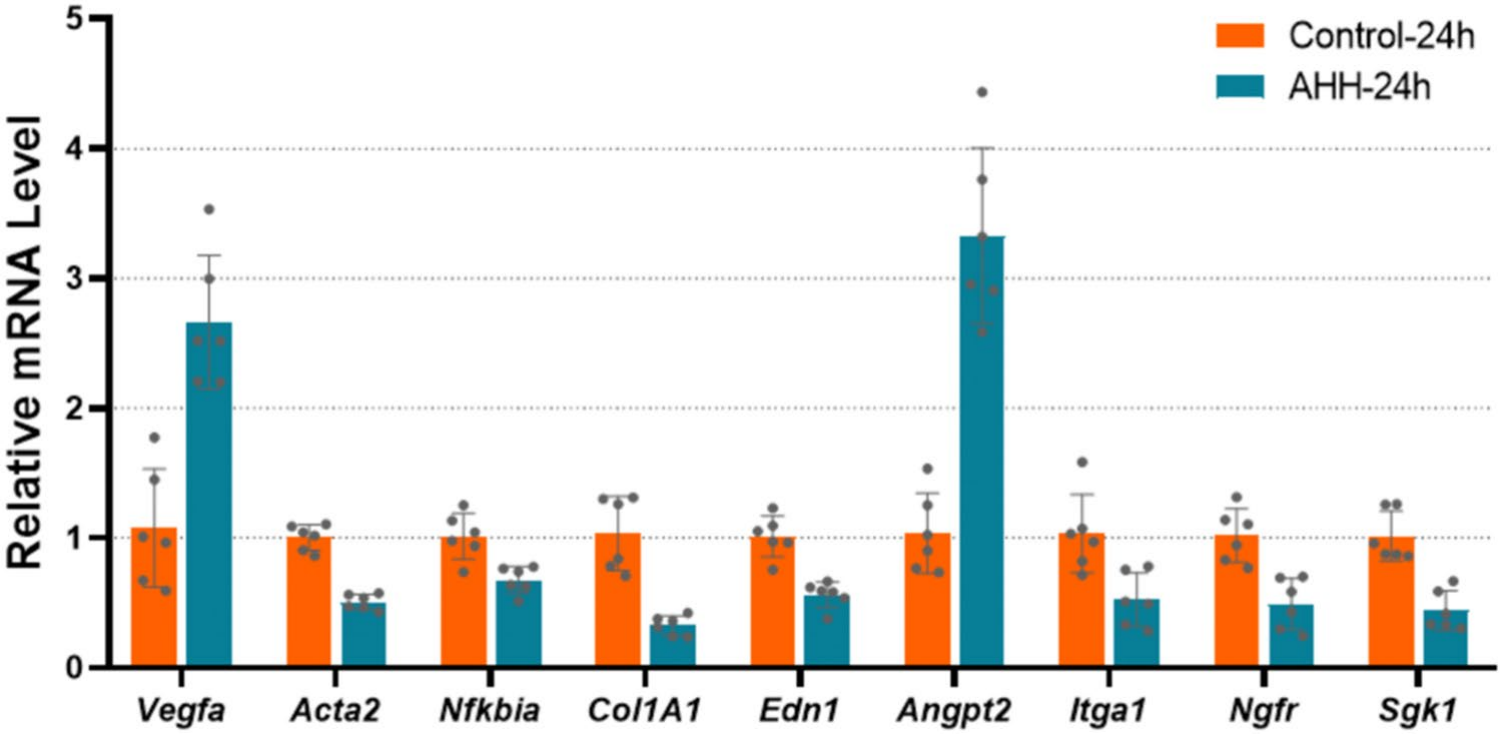
Reverse transcription-quantitative polymerase chain reaction analysis results of nine core differentially expressed genes (DEGs) screened via the protein–protein interaction network. The bars represent geometric means ± standard deviations. mRNA levels of these genes differed between the ground-level control and acute hypobaric hypoxia experimental groups (*p* < 0.05, n=6).

To validate the reliability of the transcriptome sequencing data obtained by Illumina analysis in our study, we selected nine core DEGs screened by PPI analysis for reverse transcription-qPCR using the same RNA samples. The DEGs included *Vegfa, Acta2, Nfkbia, Col1a1, Edn1*, *Angpt2*, integrin subunit alpha 1 (*Itga1*), nerve growth factor receptor (*Ngfr*), and *Sgk1* (Fig. 4 and Supplementary Table 11). The results confirmed that the RNA-seq data were reliable.

## Discussion

In this study, 112 DEGs were identified using high-throughput transcriptome sequencing analysis of samples derived from the hippocampal region of rats in the AHH experimental and control (ground-level) groups. We observed 25 upregulated and 87 down-regulated genes in the AHH group. GO, COG, and KEGG functional classification analyses were performed on the DEGs. GO functional analysis showed that the identified DEGs were primarily associated with ossification, fibrillar collagen trimer, and platelet-derived growth factor binding. Additionally, the DEGs were primarily enriched in biological process modules. In the COG functional annotation, the DEGs were classified into 18 functional categories, including general function prediction, translation, ribosomal structure and biogenesis, and replication, recombination, and repair. KEGG enrichment analysis showed that the identified DEGs were associated with the relaxin, PI3K-Akt, and amoebiasis pathways. Among the DEGs, *Vegfa*, *Acta2*, *Nfkbia*, *Col1a1*, *Edn1*, *Angpt2*, *Itga1*, *Ngfr*, and *Sgk1* play vital roles in these pathways. Inflammation plays an important role in mediating hypobaric hypoxic brain injury^7^. Furthermore, alterations in brain energy metabolism have been demonstrated in many hypoxic environments^21–24^. However, the effects of AHH on the brain have not been reported in early-stage hypertensive rats, and the involvement of inflammation and energy metabolism in this process is not well understood. Therefore, we screened for inflammation- and energy metabolism-associated DEGs by constructing PPI networks. The results revealed that both groups of genes play crucial roles in early-stage hypertension during exposure to AHH.

VEGFA was originally identified as an endothelial growth factor as well as a regulator of vascular permeability. It is produced by most cells in vivo and is significantly upregulated in response to hypoxia^25^. VEGFA induces a series of cascade responses, such as proliferation and survival, cell migration, vascular permeability, invasion of surrounding tissues, and endothelial cell inflammation, via the activation of VEGFR2^26^. VEGFA-induced expression of VEGFR2 is associated with multiple signaling pathways, including the phospholipase Cγ-extracellular regulated kinase and the PI3K-Akt pathways, both of which are closely associated with inflammation and energy metabolism^27,28^. Upregulation of VEGFA expression in the hippocampal region of SHRs under conditions involving exposure to AHH can alter the proliferation efficiency of hippocampal cells. Moreover, VEGFA, as an important vascular growth factor, affects the blood supply in the hippocampal region by altering vascular function.

*Acta2* encodes α-smooth muscle actin, which is primarily expressed in the vascular smooth muscle. Alterations in this gene are associated with several vascular diseases^29^. Changes in *Acta2* expression under conditions involving AHH may represent alterations in vasodilatory function in the hippocampus^30^.

NFKBIA is a member of a family of cellular proteins that inhibit NF-κB transcription factors. NFKBIA inhibits NF-κB by masking the nuclear localization signal of NF-κB and maintaining it in an inactive state in the cytoplasm^31^. In addition, NFKBIA can prevent NF-κB from functioning by blocking its binding to DNA^32^. NF-κB is a key regulator of pro-inflammatory gene expression, inducing the transcription of pro-inflammatory cytokines, chemokines, adhesion molecules, matrix metalloproteinases, cyclooxygenase 2, and inducible nitric oxide synthase^33^. Inhibition of NF-κB activity controls the development of inflammatory diseases^34^. Thus, overexpression of *Nfkbia* inhibits NF-κB activity and suppresses inflammatory responses in various diseases. In contrast, decreased expression of *Nfkbia* promotes the development of inflammatory responses^35^. Reduced expression of *Nfkbia* is observed in the hippocampus of SHRs under conditions involving AHH, which may result in the overactivation of inflammation-related pathways and aggravate AHH-induced cerebral damage in SHRs.

COL1A1 influences the development and prognosis of various tumors by involvement in tumor cell metastasis, proliferation, and apoptosis^36–38^. COL1A1 activates multiple signaling pathways (including epithelial-mesenchymal transition, tumor growth factor-beta, and PI3k/Akt pathways), enhances energy metabolism, promotes cell metastasis, and inhibits apoptosis^39–41^. The invasive and migratory abilities of hepatocellular carcinoma cells are significantly inhibited after the knockout of *Col1a1*^36^. In addition, the expression of the cell proliferation factor cyclin D1 and the apoptosis marker BCL-2 is decreased, whereas that of the apoptosis regulator BAX is increased after the knockdown of *Col1a1*^42^. In this study, AHH exposure resulted in the downregulation of *Colla1* expression in the hippocampal region of SHRs, which may affect normal energy metabolism and cell proliferation in the hippocampus, thereby aggravating cerebral damage.

EDN1, a strong vasoconstrictor, is closely associated with pathophysiological changes in blood vessels, and its expression is affected under hypoxic conditions^43^. Prolonged hypoxic exposure leads to elevated EDN1 expression^44^. However, upon exposure to short-term hypobaric hypoxic conditions, the expression of EDN1 is reduced in brain neurons, astrocytes, and endothelial cells^45^. This response may be a protective mechanism of brain cells against early-stage hypoxia. However, the reason why EDN1 expression is altered with prolonged hypoxia remains unknown.

ANGPT2 is an important molecule involved in the process of angiogenesis and acts as a marker of inflammation^46^. ANGPT2 levels are low under normal physiological conditions but increase during inflammation^46^. ANGPT2 acts on endothelial cells, increasing endothelial permeability. It also acts on pericytes, mediating their detachment from the basement membrane and further inducing vascular leakage^47^.

AHH can exacerbate cerebral damage in SHRs by promoting inflammation. *Itga1* encodes the integrin α1 chain, which binds to the α1 chain (ITGB1) to form a heterodimer that acts as a dual laminin/collagen receptor in neuronal and hematopoietic cells. Integrin α1 plays an important role in both fracture healing and cartilage remodeling^48,49^. The role played by *Itga1* in the hippocampal region of SHRs under AHH conditions remains to be elucidated. NGFR is a transmembrane glycoprotein. As a nerve growth factor receptor, it is involved in the mitogen-activated protein kinase, Ras, PI3K-Akt, and the apoptosis signaling pathways in several species^50–53^. NGFR expression is closely associated with cell growth, proliferation, and apoptosis. *Sgk1* is transcriptionally regulated by serum and glucocorticoids^54^. SGK1 activates several ion channels, transporter proteins, transcription factors, and enzymes^50^, and its expression is strongly upregulated in a variety of cardiovascular diseases, which are closely associated with vascular calcification^55,56^. Downregulation of *Sgk1* alleviates inflammation via inhibition of the NF-κB signaling pathway^57^. However, *Sgk1* knockdown reduces the potency of protective mechanisms associated with hypoxia/reoxygenation injury in cardiomyocytes^58^. Therefore, the effects attributed to the downregulation of *Sgk1* expression in AHH should be further investigated in SHRs.

Our study provides the first demonstration of the significant influence of AHH exposure on gene expression changes in the hippocampal region during early spontaneous hypertension in rats. Furthermore, our results showed that energy metabolism and inflammation play important roles in early-stage hypertension under conditions involving AHH.

This study has few limitations. First, the experimental conditions employed were limited. We used six-week-old SHRs exposed to AHH to examine the changes in gene expression in the hippocampal region of rats in the early stages of hypertension. The next step would be to select SHRs of different ages to investigate the gene expression changes in the hippocampal region during intermediate and late-stage hypertension upon AHH exposure. Alternatively, Wistar Kyoto rats can be used as experimental animals to exclude the effect of AHH exposure in normal rats. Furthermore, we have performed RNA-seq and qPCR validation of selected genes to determine the alterations in the expression of related genes in the hippocampal region of the early-stage SHR model under AHH exposure. Follow-up studies are necessary to compare the effects of AHH exposure on hypertensive brain injury in different periods by increasing the sample size and further experimental grouping. Additionally, the experimental analyses should be enriched for a more in-depth study of the alterations in the associated genes.

## Supplementary Information

Supplementary Information 1.

Supplementary Information 2.

Supplementary Information 3.

Supplementary Information 4.

Supplementary Information 5.

Supplementary Information 6.

Supplementary Information 7.

Supplementary Information 8.

Supplementary Information 9.

Supplementary Information 10.

Supplementary Information 11.

## Data Availability

The datasets generated and/or analyzed during the current study are available in the NCBI—SRA (Sequence Read Archive) repository, accession number: PRJNA891503.
