# Supplementary material for: Transcriptomic analysis of the cerebral hippocampal tissue in spontaneously hypertensive rats exposed to acute hypobaric hypoxia: associations with inflammation and energy metabolism
Source: Sci Rep. 2023 Mar 6;13:3681. doi: 10.1038/s41598-023-30682-0 (PMC9988845; doi:10.1038/s41598-023-30682-0)
Supplement: Supplementary file 1 — Supplementary Information 1. [file 41598_2023_30682_MOESM1_ESM.pdf]

Table S1. The blood pressure of spontaneously hypertensive rats.

Sample Name	SBP(mmHg)					DBP(mmHg)				
Control-24h-1	153	148	151	158	153	124	115	116	109	106
Control-24h-2	159	143	150	155	145	110	108	112	113	105
Control-24h-3	144	142	140	147	146	108	106	111	105	103
Control-24h-4	160	155	149	152	159	113	119	128	112	115
Control-24h-5	156	156	157	159	160	103	110	112	110	108
Control-24h-6	150	151	155	154	153	111	115	116	114	110
Control-24h-7	151	156	145	148	148	100	101	99	104	102
Control-24h-8	145	150	148	148	146	101	103	101	103	104
Control-24h-9	148	149	150	154	151	102	109	103	101	105
AHH-24h-1	156	152	150	149	151	127	119	114	110	108
AHH-24h-2	146	149	150	151	147	108	110	108	104	106
AHH-24h-3	150	151	144	152	149	119	114	107	113	114
AHH-24h-4	155	152	151	145	151	111	109	115	110	113
AHH-24h-5	149	148	150	148	151	101	108	102	103	105
AHH-24h-6	161	159	158	155	158	159	116	118	117	114
AHH-24h-7	154	155	154	153	151	110	108	109	106	104
AHH-24h-8	153	148	151	153	153	107	109	104	103	106
AHH-24h-9	159	155	153	154	151	109	103	105	106	104

SBP: systolic blood pressure. DBP: diastolic blood pressure.
