# Supplementary material for: Transcriptomic analysis of the cerebral hippocampal tissue in spontaneously hypertensive rats exposed to acute hypobaric hypoxia: associations with inflammation and energy metabolism
Source: Sci Rep. 2023 Mar 6;13:3681. doi: 10.1038/s41598-023-30682-0 (PMC9988845; doi:10.1038/s41598-023-30682-0)
Supplement: Supplementary file 2 — Supplementary Information 2. [file 41598_2023_30682_MOESM2_ESM.pdf]

Table S10. Summary of Kyoto Encyclopedia of Genes and Genomes (KEGG) top 10 pathways analysis of differential expression genes (DEGs).

Pathway ID	Description	Number of DEGs in category
rno04926	Relaxin signaling pathway	7
rno04151	PI3K-Akt signaling pathway	9
rno04014	Ras signaling pathway	6
rno04064	NF-kappa B signaling pathway	4
rno04933	AGE-RAGE signaling pathway in diabetic complications	4
rno04010	MAPK signaling pathway	6
rno04150	mTOR signaling pathway	4
rno04310	Wnt signaling pathway	4
rno04066	HIF-1 signaling pathway	3
rno04722	Neurotrophin signaling pathway	3
