# Supplementary material for: Transcriptomic analysis of the cerebral hippocampal tissue in spontaneously hypertensive rats exposed to acute hypobaric hypoxia: associations with inflammation and energy metabolism
Source: Sci Rep. 2023 Mar 6;13:3681. doi: 10.1038/s41598-023-30682-0 (PMC9988845; doi:10.1038/s41598-023-30682-0)
Supplement: Supplementary file 3 — Supplementary Information 3. [file 41598_2023_30682_MOESM3_ESM.pdf]

Table S11. Reverse transcription qPCR data for 9 differential expression genes.

Vegfa

Simple	Control-24h-4	Control-24h-5	Control-24h-6	Control-24h-7	Control-24h-8	Control-24h-9	AHH-24h-4	AHH-24h-5	AHH-24h-6	AHH-24h-7	AHH-24h-8	AHH-24h-9
ΔCT	6.35272	7.45879	6.87563	6.06004	7.63816	6.93792	5.066	5.748	5.745	5.304	5.556	5.555
F	1.44843	0.67288	1.00806	1.7742	0.59421	0.96546	3.534	2.202	2.207	2.997	2.517	2.519
Mean			1.077207692						2.662441222			
SD			0.415861998						0.471237078			

Acta2

Simple	Control-24h-4	Control-24h-5	Control-24h-6	Control-24h-7	Control-24h-8	Control-24h-9	AHH-24h-4	AHH-24h-5	AHH-24h-6	AHH-24h-7	AHH-24h-8	AHH-24h-9
ΔCT	4.0292	4.36637	4.12545	4.09059	4.00846	4.29452	5.049	4.952	5.235	4.983	5.372	5.26
F	1.08917	0.86218	1.01888	1.0438	1.10494	0.90621	0.537	0.575	0.472	0.562	0.43	0.464
Mean			1.004196202						0.506592542			
SD			0.090300582						0.054127346			

Nfkbia

Simple	Control-24h-4	Control-24h-5	Control-24h-6	Control-24h-7	Control-24h-8	Control-24h-9	AHH-24h-4	AHH-24h-5	AHH-24h-6	AHH-24h-7	AHH-24h-8	AHH-24h-9
ΔCT	3.77481	3.71469	4.1205	3.50285	3.35678	3.6229	4.112	4.65	4.325	4.046	4.07	4.35
F	0.93775	0.97766	0.73795	1.13228	1.25293	1.04188	0.743	0.511	0.64	0.777	0.764	0.63
Mean			1.013408664						0.677510765			
SD			0.160828992						0.094007537			

Col1A1

Simple	Control-24h-4	Control-24h-5	Control-24h-6	Control-24h-7	Control-24h-8	Control-24h-9	AHH-24h-4	AHH-24h-5	AHH-24h-6	AHH-24h-7	AHH-24h-8	AHH-24h-9
ΔCT	5.78789	5.77386	5.83255	6.51594	6.41595	6.66828	7.641	7.412	8.223	7.575	7.832	8.248
F	1.29941	1.31211	1.2598	0.78448	0.84078	0.70586	0.36	0.421	0.24	0.377	0.315	0.236
Mean			1.033739313						0.324866558			
SD			0.260140655						0.068727245			

Edn1

Simple	Control-24h-4	Control-24h-5	Control-24h-6	Control-24h-7	Control-24h-8	Control-24h-9	AHH-24h-4	AHH-24h-5	AHH-24h-6	AHH-24h-7	AHH-24h-8	AHH-24h-9
ΔCT	7.28287	7.33799	7.11432	7.46261	7.45657	7.81616	8	8.102	8.822	8.307	8.168	8.192
F	1.09344	1.05246	1.22896	0.96536	0.96941	0.75555	0.665	0.62	0.376	0.538	0.592	0.582
Mean			1.01086414						0.562217983			
SD			0.144389619						0.091652558			

Angpt2

Simple	Control-24h-4	Control-24h-5	Control-24h-6	Control-24h-7	Control-24h-8	Control-24h-9	AHH-24h-4	AHH-24h-5	AHH-24h-6	AHH-24h-7	AHH-24h-8	AHH-24h-9
ΔCT	8.37199	9.13723	9.4352	8.66467	8.95786	9.37291	7.078	7.258	7.427	6.84	7.45	7.618
F	1.53473	0.90297	0.73447	1.25293	1.02251	0.76688	3.763	3.323	2.954	4.437	2.907	2.589
Mean			1.035747912						3.328774783			
SD			0.281962069						0.616700484			

Itga1

Simple	Control-24h-4	Control-24h-5	Control-24h-6	Control-24h-7	Control-24h-8	Control-24h-9	AHH-24h-4	AHH-24h-5	AHH-24h-6	AHH-24h-7	AHH-24h-8	AHH-24h-9

**ΔCT**	5. 50999	5. 31168	4. 92643	5.07293	4.98417	4.36192	5. 385	6. 002	6. 865	6.621	5.431	6.046
**F**	0.71592	0.82141	1.07283	0.96924	1.03074	1.5866	0.781	0.509	0.28	0.332	0.756	0.494
**Mean**			1.032789003						0.525185989			
**SD**			0.276116493						0.190448448			

Ngfr

**Simple**	**Control-24h-4**	**Control-24h-5**	**Control-24h-6**	**Control-24h-7**	**Control-24h-8**	**Control-24h-9**	**AHH-24h-4**	**AHH-24h-5**	**AHH-24h-6**	**AHH-24h-7**	**AHH-24h-8**	**AHH-24h-9**
**ΔCT**	10. 3978	10. 8095	10. 919	10.6274	10.1487	10.3544	11. 05	11. 31	12. 58	12.29	11.07	11.76
**F**	1.10574	0.8312	0.77049	0.94305	1.31413	1.13947	0.702	0.587	0.244	0.298	0.692	0.43
**Mean**			1.01734611						0.492148205			
**SD**			0.187934565						0.180826636			

Sgk1

**Simple**	**Control-24h-4**	**Control-24h-5**	**Control-24h-6**	**Control-24h-7**	**Control-24h-8**	**Control-24h-9**	**AHH-24h-4**	**AHH-24h-5**	**AHH-24h-6**	**AHH-24h-7**	**AHH-24h-8**	**AHH-24h-9**
**ΔCT**	3. 84672	3. 81659	3. 8232	3.29841	3.29308	3.68931	5. 35	5. 229	5. 209	4.389	4.209	4.888
**F**	0.85926	0.87739	0.87338	1.25656	1.26121	0.95832	0.303	0.33	0.334	0.59	0.669	0.418
**Mean**			1.014352459						0.440479918			
**SD**			0.175796019						0.13990341			

Sample name: sample name of the animal from different groups. ΔCT = CT(target, test) – CT(ref, test). $F = 2^{-\Delta\Delta CT} = 2^{-\Delta CT(Simple1) - \Delta CT(Calibrator1)}$. Mean: mean of F value from the same group. SD: standard deviation of F value from the same group.
