# Supplementary material for: Transcriptomic analysis of the cerebral hippocampal tissue in spontaneously hypertensive rats exposed to acute hypobaric hypoxia: associations with inflammation and energy metabolism
Source: Sci Rep. 2023 Mar 6;13:3681. doi: 10.1038/s41598-023-30682-0 (PMC9988845; doi:10.1038/s41598-023-30682-0)
Supplement: Supplementary file 4 — Supplementary Information 4. [file 41598_2023_30682_MOESM4_ESM.pdf]

Table S2. Primer sequences for RT-qPCR.

Gene		Primer sequence	prodSize
vegfa	FORWARD	CACCAAAGCCAGCACATAGGAGAG	144bp
	REVERSE	CTGCGGATCTTGGACAAACAAATGC	
Acta2	FORWARD	GCGTGGCTATTCCTTCGTGACTAC	149bp
	REVERSE	CATCAGGCAGTTCGTAGCTCTTCTC	
Nfkbia	FORWARD	ACTTGGTGACTTTGGGTGCTGATG	118bp
	REVERSE	CCACACTTCAACAGGAGCGAGAC	
Col1a1	FORWARD	TGTTGGTCCTGCTGGCAAGAATG	145bp
	REVERSE	GTCACCTTGTTCGCCTGTCTCAC	
Edn1	FORWARD	CTTCTGCCACCTGGACATCATCTG	132bp
	REVERSE	CTGTTCCCTTGGTCTGTGGTCTTTG	
Angpt2	FORWARD	CAGTAGCATCAGCCAACCAGGAAG	114bp
	REVERSE	CCACATGCGTCGAACCACCAG	
Itga1	FORWARD	CAAGTGAATGTCTCGCTCCTCCTG	136bp
	REVERSE	CTCGCTTCCGGTTGCTGCTAC	
Ngfr	FORWARD	GCTGCTGCTGATTCTAGGGATGTC	150bp
	REVERSE	GGTTCACACACGGTCTGGTTGG	
Sgk1	FORWARD	GCCAAACCCTCTGACTTCCACTTC	90bp
	REVERSE	TGCTTCTTCTGCCTTGTGCCTTG	
β-Actin	FORWARD	GAAGTGTGACGTTGACATCCG	282bp
	REVERSE	GCCTAGAAGCATTTGCGGTG	

Primer sequences were retrieved from PrimerBank 40, and primer specificity was further validated using the National Center for Biotechnology Information Primer-BLAST tool.
