# Supplementary material for: Transcriptomic analysis of the cerebral hippocampal tissue in spontaneously hypertensive rats exposed to acute hypobaric hypoxia: associations with inflammation and energy metabolism
Source: Sci Rep. 2023 Mar 6;13:3681. doi: 10.1038/s41598-023-30682-0 (PMC9988845; doi:10.1038/s41598-023-30682-0)
Supplement: Supplementary file 5 — Supplementary Information 5. [file 41598_2023_30682_MOESM5_ESM.pdf]

Table S3. Summary of Gene Ontology (GO) analysis of 112 differential expression genes (DEGs).

Term Type	GO ID	Term description	Count
Biological_process	GO:0001503	ossification	13
Biological_process	GO:0001569	branching involved in blood vessel morphogenesis	5
Biological_process	GO:0031214	biomineral tissue development	8
Biological_process	GO:0110148	biomineralization	8
Biological_process	GO:0007411	axon guidance	9
Biological_process	GO:0097485	neuron projection guidance	9
Biological_process	GO:0031960	response to corticosteroid	10
Biological_process	GO:0009612	response to mechanical stimulus	10
Biological_process	GO:0030199	collagen fibril organization	5
Biological_process	GO:0060343	trabecula formation	4
Biological_process	GO:0007271	synaptic transmission, cholinergic	4
Biological_process	GO:0051384	response to glucocorticoid	9
Biological_process	GO:0009266	response to temperature stimulus	8
Biological_process	GO:0048841	regulation of axon extension involved in axon guidance	4
Biological_process	GO:0071385	cellular response to glucocorticoid stimulus	6
Biological_process	GO:0003018	vascular process in circulatory system	8
Biological_process	GO:0060346	bone trabecula formation	3
Biological_process	GO:0071356	cellular response to tumor necrosis factor	8
Biological_process	GO:0030282	bone mineralization	6
Biological_process	GO:0048846	axon extension involved in axon guidance	4
Biological_process	GO:1902284	neuron projection extension involved in neuron projection guidance	4
Biological_process	GO:0071384	cellular response to corticosteroid stimulus	6
Biological_process	GO:0071559	response to transforming growth factor beta	8
Biological_process	GO:0034612	response to tumor necrosis factor	8
Biological_process	GO:0061430	bone trabecula morphogenesis	3

Biological_process	GO:0070431	nucleotide-binding oligomerization domain containing 2 signaling pathway	3
Biological_process	GO:0001961	positive regulation of cytokine-mediated signaling pathway	4
Biological_process	GO:0060485	mesenchyme development	8
Biological_process	GO:0070423	nucleotide-binding oligomerization domain containing signaling pathway	3
Biological_process	GO:0035872	nucleotide-binding domain, leucine rich repeat containing receptor signaling pathway	3
Biological_process	GO:0007409	axonogenesis	10
Biological_process	GO:0048762	mesenchymal cell differentiation	7
Biological_process	GO:0097191	extrinsic apoptotic signaling pathway	7
Biological_process	GO:0060760	positive regulation of response to cytokine stimulus	4
Biological_process	GO:0061383	trabecula morphogenesis	4
Biological_process	GO:0072593	reactive oxygen species metabolic process	7
Biological_process	GO:0001755	neural crest cell migration	4
Biological_process	GO:0007588	excretion	4
Biological_process	GO:0044070	regulation of anion transport	5
Biological_process	GO:0001558	regulation of cell growth	9
Biological_process	GO:0048754	branching morphogenesis of an epithelial tube	6
Biological_process	GO:0071560	cellular response to transforming growth factor beta stimulus	7
Biological_process	GO:0006939	smooth muscle contraction	5
Biological_process	GO:0050926	regulation of positive chemotaxis	3
Biological_process	GO:0001667	ameboidal-type cell migration	9
Biological_process	GO:0015837	amine transport	5
Biological_process	GO:0014829	vascular associated smooth muscle contraction	3
Biological_process	GO:0035809	regulation of urine volume	3
Biological_process	GO:0006940	regulation of smooth muscle contraction	4
Biological_process	GO:0003014	renal system process	5

Biological_process	GO:0060384	innervation	3
Biological_process	GO:0030278	regulation of ossification	5
Biological_process	GO:0015711	organic anion transport	8
Biological_process	GO:0035094	response to nicotine	4
Biological_process	GO:0045123	cellular extravasation	4
Biological_process	GO:0038034	signal transduction in absence of ligand	4
Biological_process	GO:0097192	extrinsic apoptotic signaling pathway in absence of ligand	4
Biological_process	GO:0045987	positive regulation of smooth muscle contraction	3
Biological_process	GO:0060070	canonical Wnt signaling pathway	7
Biological_process	GO:0060562	epithelial tube morphogenesis	8
Biological_process	GO:0008217	regulation of blood pressure	6
Biological_process	GO:0030218	erythrocyte differentiation	5
Biological_process	GO:1901099	negative regulation of signal transduction in absence of ligand	3
Biological_process	GO:2001240	negative regulation of extrinsic apoptotic signaling pathway in absence of ligand	3
Biological_process	GO:0061448	connective tissue development	7
Biological_process	GO:0051216	cartilage development	6
Biological_process	GO:0046942	carboxylic acid transport	7
Biological_process	GO:0033209	tumor necrosis factor-mediated signaling pathway	4
Biological_process	GO:0071542	dopaminergic neuron differentiation	3
Biological_process	GO:0001649	osteoblast differentiation	6
Biological_process	GO:0048675	axon extension	5
Biological_process	GO:2001233	regulation of apoptotic signaling pathway	8
Biological_process	GO:0061138	morphogenesis of a branching epithelium	6
Biological_process	GO:0001837	epithelial to mesenchymal transition	5
Biological_process	GO:0008361	regulation of cell size	6
Biological_process	GO:0019221	cytokine-mediated signaling pathway	8
Biological_process	GO:0014032	neural crest cell development	4

Biological_process	GO:0042476	odontogenesis	5
Biological_process	GO:0034101	erythrocyte homeostasis	5
Biological_process	GO:0035811	negative regulation of urine volume	2
Biological_process	GO:0098700	neurotransmitter loading into synaptic vesicle	2
Biological_process	GO:1901029	negative regulation of mitochondrial outer membrane permeabilization involved in apoptotic signaling pathway	2
Biological_process	GO:0002687	positive regulation of leukocyte migration	5
Biological_process	GO:0015695	organic cation transport	3
Biological_process	GO:0055067	monovalent inorganic cation homeostasis	5
Biological_process	GO:0050920	regulation of chemotaxis	6
Biological_process	GO:0010717	regulation of epithelial to mesenchymal transition	4
Biological_process	GO:0014031	mesenchymal cell development	4
Biological_process	GO:0048864	stem cell development	4
Biological_process	GO:0048863	stem cell differentiation	6
Biological_process	GO:0002691	regulation of cellular extravasation	3
Biological_process	GO:0030099	myeloid cell differentiation	8
Biological_process	GO:0016055	Wnt signaling pathway	8
Biological_process	GO:0010968	regulation of microtubule nucleation	2
Biological_process	GO:0060174	limb bud formation	2
Biological_process	GO:0070424	regulation of nucleotide-binding oligomerization domain containing signaling pathway	2
Biological_process	GO:0090084	negative regulation of inclusion body assembly	2
Biological_process	GO:0021675	nerve development	4
Biological_process	GO:0043270	positive regulation of ion transport	7
Biological_process	GO:0060323	head morphogenesis	3
Biological_process	GO:0198738	cell-cell signaling by wnt	8
Biological_process	GO:0001763	morphogenesis of a branching structure	6
Biological_process	GO:0014033	neural crest cell differentiation	4

Biological_process	GO:0031110	regulation of microtubule polymerization or depolymerization	4
Biological_process	GO:0015849	organic acid transport	7
Biological_process	GO:0072006	nephron development	5
Biological_process	GO:0085029	extracellular matrix assembly	3
Biological_process	GO:2001236	regulation of extrinsic apoptotic signaling pathway	5
Biological_process	GO:0042088	T-helper 1 type immune response	3
Biological_process	GO:0045117	azole transmembrane transport	2
Biological_process	GO:0070472	regulation of uterine smooth muscle contraction	2
Biological_process	GO:1903265	positive regulation of tumor necrosis factor-mediated signaling pathway	2
Biological_process	GO:0070167	regulation of biomineral tissue development	4
Biological_process	GO:2001237	negative regulation of extrinsic apoptotic signaling pathway	4
Biological_process	GO:0022407	regulation of cell-cell adhesion	8
Biological_process	GO:0110149	regulation of biomineralization	4
Biological_process	GO:0035107	appendage morphogenesis	5
Biological_process	GO:0035108	limb morphogenesis	5
Biological_process	GO:0048705	skeletal system morphogenesis	6
Biological_process	GO:0071383	cellular response to steroid hormone stimulus	6
Biological_process	GO:0032905	transforming growth factor beta1 production	2
Biological_process	GO:0043589	skin morphogenesis	2
Biological_process	GO:0048251	elastic fiber assembly	2
Biological_process	GO:0060179	male mating behavior	2
Biological_process	GO:0070471	uterine smooth muscle contraction	2
Biological_process	GO:0010718	positive regulation of epithelial to mesenchymal transition	3
Biological_process	GO:2001239	regulation of extrinsic apoptotic signaling pathway in absence of ligand	3
Biological_process	GO:0071548	response to dexamethasone	4

Biological_process	GO:0002753	cytoplasmic pattern recognition receptor signaling pathway	3
Biological_process	GO:0045933	positive regulation of muscle contraction	3
Biological_process	GO:0016525	negative regulation of angiogenesis	4
Biological_process	GO:0043618	regulation of transcription from RNA polymerase II promoter in response to stress	3
Biological_process	GO:0045124	regulation of bone resorption	3
Biological_process	GO:0007179	transforming growth factor beta receptor signaling pathway	5
Biological_process	GO:0035296	regulation of tube diameter	5
Biological_process	GO:0097746	blood vessel diameter maintenance	5
Biological_process	GO:0036293	response to decreased oxygen levels	8
Biological_process	GO:0035150	regulation of tube size	5
Biological_process	GO:0051954	positive regulation of amine transport	3
Biological_process	GO:0002027	regulation of heart rate	4
Biological_process	GO:2000181	negative regulation of blood vessel morphogenesis	4
Biological_process	GO:0001822	kidney development	7
Biological_process	GO:1903522	regulation of blood circulation	6
Biological_process	GO:1901343	negative regulation of vasculature development	4
Biological_process	GO:0010039	response to iron ion	3
Biological_process	GO:0035815	positive regulation of renal sodium excretion	2
Biological_process	GO:0060700	regulation of ribonuclease activity	2
Biological_process	GO:0097201	negative regulation of transcription from RNA polymerase II promoter in response to stress	2
Biological_process	GO:1903587	regulation of blood vessel endothelial cell proliferation involved in sprouting angiogenesis	2
Biological_process	GO:0060537	muscle tissue development	8
Biological_process	GO:0000302	response to reactive oxygen species	6
Biological_process	GO:0060840	artery development	4
Biological_process	GO:0032892	positive regulation of organic acid transport	3

Biological_process	GO:0043620	regulation of DNA-templated transcription in response to stress	3
Biological_process	GO:0042310	vasoconstriction	4
Biological_process	GO:0045666	positive regulation of neuron differentiation	4
Biological_process	GO:0072132	mesenchyme morphogenesis	3
Biological_process	GO:0001764	neuron migration	5
Biological_process	GO:0030307	positive regulation of cell growth	5
Biological_process	GO:0032308	positive regulation of prostaglandin secretion	2
Biological_process	GO:0030516	regulation of axon extension	4
Biological_process	GO:0050900	leukocyte migration	7
Biological_process	GO:1904645	response to amyloid-beta	3
Biological_process	GO:0072001	renal system development	7
Biological_process	GO:0001101	response to acid chemical	6
Biological_process	GO:0002262	myeloid cell homeostasis	5
Biological_process	GO:1903793	positive regulation of anion transport	3
Biological_process	GO:0030879	mammary gland development	5
Biological_process	GO:0051897	positive regulation of protein kinase B signaling	4
Biological_process	GO:0051952	regulation of amine transport	4
Biological_process	GO:0042756	drinking behavior	2
Biological_process	GO:0045820	negative regulation of glycolytic process	2
Biological_process	GO:0050930	induction of positive chemotaxis	2
Biological_process	GO:0001959	regulation of cytokine-mediated signaling pathway	4
Biological_process	GO:1902903	regulation of supramolecular fiber organization	7
Biological_process	GO:0010171	body morphogenesis	3
Biological_process	GO:0015718	monocarboxylic acid transport	4
Biological_process	GO:0031113	regulation of microtubule polymerization	3
Biological_process	GO:0048736	appendage development	5
Biological_process	GO:0060173	limb development	5
Biological_process	GO:0050922	negative regulation of chemotaxis	3

Biological_process	GO:0031109	microtubule polymerization or depolymerization	4
Biological_process	GO:0071774	response to fibroblast growth factor	4
Biological_process	GO:0021872	forebrain generation of neurons	3
Biological_process	GO:0046850	regulation of bone remodeling	3
Biological_process	GO:0055078	sodium ion homeostasis	3
Biological_process	GO:1990138	neuron projection extension	5
Biological_process	GO:0045765	regulation of angiogenesis	6
Biological_process	GO:0032306	regulation of prostaglandin secretion	2
Biological_process	GO:0033605	positive regulation of catecholamine secretion	2
Biological_process	GO:0042026	protein refolding	2
Biological_process	GO:0043252	sodium-independent organic anion transport	2
Biological_process	GO:0071389	cellular response to mineralocorticoid stimulus	2
Biological_process	GO:0090083	regulation of inclusion body assembly	2
Biological_process	GO:1901673	regulation of mitotic spindle assembly	2
Biological_process	GO:0007162	negative regulation of cell adhesion	6
Biological_process	GO:1902905	positive regulation of supramolecular fiber organization	5
Biological_process	GO:0046717	acid secretion	3
Cellular_component	GO:0005583	fibrillar collagen trimer	5
Cellular_component	GO:0098643	banded collagen fibril	5
Cellular_component	GO:0098644	complex of collagen trimers	5
Cellular_component	GO:0062023	collagen-containing extracellular matrix	9
Cellular_component	GO:0031012	extracellular matrix	11
Cellular_component	GO:0030312	external encapsulating structure	11
Cellular_component	GO:0005581	collagen trimer	5
Cellular_component	GO:0030141	secretory granule	10
Cellular_component	GO:0045121	membrane raft	10
Cellular_component	GO:0098857	membrane microdomain	10
Cellular_component	GO:0045334	clathrin-coated endocytic vesicle	3
Cellular_component	GO:0030136	clathrin-coated vesicle	5

Cellular_component	GO:0043235	receptor complex	8
Cellular_component	GO:0045178	basal part of cell	7
Cellular_component	GO:0098802	plasma membrane signaling receptor complex	5
Cellular_component	GO:0009898	cytoplasmic side of plasma membrane	5
Cellular_component	GO:0005892	acetylcholine-gated channel complex	2
Cellular_component	GO:0098981	cholinergic synapse	2
Cellular_component	GO:0030135	coated vesicle	5
Cellular_component	GO:0098562	cytoplasmic side of membrane	5
Cellular_component	GO:0009897	external side of plasma membrane	7
Molecular_function	GO:0048407	platelet-derived growth factor binding	4
Molecular_function	GO:0019838	growth factor binding	8
Molecular_function	GO:0005201	extracellular matrix structural constituent	5
Molecular_function	GO:0048018	receptor ligand activity	10
Molecular_function	GO:0030546	signaling receptor activator activity	10
Molecular_function	GO:0002020	protease binding	6
Molecular_function	GO:0001664	G protein-coupled receptor binding	8
Molecular_function	GO:0030545	signaling receptor regulator activity	10
Molecular_function	GO:0005125	cytokine activity	6
Molecular_function	GO:0015101	organic cation transmembrane transporter activity	3
Molecular_function	GO:0008509	anion transmembrane transporter activity	7
Molecular_function	GO:1901474	azole transmembrane transporter activity	2
Molecular_function	GO:0005452	inorganic anion exchanger activity	2
Molecular_function	GO:0042166	acetylcholine binding	2
Molecular_function	GO:0008514	organic anion transmembrane transporter activity	5
Molecular_function	GO:0005161	platelet-derived growth factor receptor binding	2
Molecular_function	GO:1904315	transmitter-gated ion channel activity involved in regulation of postsynaptic membrane potential	3
Molecular_function	GO:0031005	filamin binding	2

Molecular_function	GO:0099529	neurotransmitter receptor activity involved in regulation of postsynaptic membrane potential	3
Molecular_function	GO:0050839	cell adhesion molecule binding	6
Molecular_function	GO:0022848	acetylcholine-gated cation-selective channel activity	2
Molecular_function	GO:0022824	transmitter-gated ion channel activity	3
Molecular_function	GO:0022835	transmitter-gated channel activity	3
Molecular_function	GO:0015347	sodium-independent organic anion transmembrane transporter activity	2
Molecular_function	GO:0015464	acetylcholine receptor activity	2
Molecular_function	GO:0005179	hormone activity	4
Molecular_function	GO:0005230	extracellular ligand-gated ion channel activity	3
Molecular_function	GO:0098960	postsynaptic neurotransmitter receptor activity	3
Molecular_function	GO:0015103	inorganic anion transmembrane transporter activity	4
