# Supplementary material for: Transcriptomic analysis of the cerebral hippocampal tissue in spontaneously hypertensive rats exposed to acute hypobaric hypoxia: associations with inflammation and energy metabolism
Source: Sci Rep. 2023 Mar 6;13:3681. doi: 10.1038/s41598-023-30682-0 (PMC9988845; doi:10.1038/s41598-023-30682-0)
Supplement: Supplementary file 6 — Supplementary Information 6. [file 41598_2023_30682_MOESM6_ESM.pdf]

Table S4. The table of up-regulated differentially expressed genes (DEGs) using Gene Ontology (GO) terms annotation cluster.

Term Type	Term description	Number of up-regulated DEGs
Biological_process	regulation of cell-cell adhesion	6
Biological_process	positive regulation of cell adhesion	5
Biological_process	positive regulation of cell-cell adhesion	4
Biological_process	regulation of alpha-beta T cell activation	3
Biological_process	regulation of leukocyte cell-cell adhesion	4
Biological_process	regulation of T cell activation	4
Biological_process	erythrocyte differentiation	3
Biological_process	leukocyte cell-cell adhesion	4
Biological_process	regulation of positive chemotaxis	2
Biological_process	erythrocyte homeostasis	3
Biological_process	alpha-beta T cell activation	3
Biological_process	regulation of endothelial cell migration	3
Biological_process	regulation of T cell proliferation	3
Biological_process	regulation of alpha-beta T cell proliferation	2
Biological_process	erythrocyte development	2
Biological_process	positive regulation of morphogenesis of an epithelium	2
Biological_process	alpha-beta T cell proliferation	2
Biological_process	myeloid cell homeostasis	3
Biological_process	response to hydrogen peroxide	3
Biological_process	regulation of lymphocyte activation	4
Biological_process	positive regulation of T cell activation	3
Biological_process	positive regulation of alpha-beta T cell differentiation	2
Biological_process	T cell proliferation	3
Biological_process	endothelial cell migration	3

Biological_process	positive regulation of leukocyte cell-cell adhesion	3
Biological_process	extrinsic apoptotic signaling pathway	3
Biological_process	regulation of cell-substrate adhesion	3
Biological_process	regulation of lymphocyte proliferation	3
Biological_process	regulation of epithelial cell migration	3
Biological_process	regulation of mononuclear cell proliferation	3
Cellular_component	cytoplasmic side of plasma membrane	3
Cellular_component	cytoplasmic side of membrane	3
