# Supplementary material for: Transcriptomic analysis of the cerebral hippocampal tissue in spontaneously hypertensive rats exposed to acute hypobaric hypoxia: associations with inflammation and energy metabolism
Source: Sci Rep. 2023 Mar 6;13:3681. doi: 10.1038/s41598-023-30682-0 (PMC9988845; doi:10.1038/s41598-023-30682-0)
Supplement: Supplementary file 7 — Supplementary Information 7. [file 41598_2023_30682_MOESM7_ESM.pdf]

Table S5. The table of down-regulated differentially expressed genes (DEGs) using Gene Ontology (GO) terms annotation cluster.

Term Type	Term description	Number of down-regulated DEGs
Biological_process	collagen fibril organization	5
Biological_process	axon guidance	8
Biological_process	neuron projection guidance	8
Biological_process	response to corticosteroid	9
Biological_process	ossification	10
Biological_process	synaptic transmission, cholinergic	4
Biological_process	response to mechanical stimulus	9
Biological_process	response to transforming growth factor beta	8
Biological_process	branching involved in blood vessel morphogenesis	4
Biological_process	response to glucocorticoid	8
Biological_process	nucleotide-binding oligomerization domain containing 2 signaling pathway	3
Biological_process	positive regulation of cytokine-mediated signaling pathway	4
Biological_process	response to temperature stimulus	7
Biological_process	vascular process in circulatory system	7
Biological_process	nucleotide-binding oligomerization domain containing signaling pathway	3
Biological_process	biomineral tissue development	6
Biological_process	biomineralization	6
Biological_process	cellular response to tumor necrosis factor	7
Biological_process	nucleotide-binding domain, leucine rich repeat containing receptor signaling pathway	3
Biological_process	positive regulation of response to cytokine stimulus	4

Biological_process	cellular response to transforming growth factor beta stimulus	7
Biological_process	neural crest cell migration	4
Biological_process	excretion	4
Biological_process	regulation of anion transport	5
Biological_process	cellular response to glucocorticoid stimulus	5
Biological_process	response to tumor necrosis factor	7
Biological_process	axonogenesis	9
Biological_process	smooth muscle contraction	5
Biological_process	amine transport	5
Biological_process	mesenchyme development	7
Biological_process	cellular response to corticosteroid stimulus	5
Biological_process	canonical Wnt signaling pathway	7
Biological_process	regulation of smooth muscle contraction	4
Biological_process	trabecula formation	3
Biological_process	carboxylic acid transport	7
Biological_process	vascular associated smooth muscle contraction	3
Biological_process	regulation of blood pressure	6
Biological_process	response to nicotine	4
Biological_process	cellular extravasation	4
Biological_process	innervation	3
Biological_process	Wnt signaling pathway	8
Biological_process	cell-cell signaling by wnt	8
Biological_process	regulation of axon extension involved in axon guidance	3
Biological_process	mesenchymal cell differentiation	6
Biological_process	tumor necrosis factor-mediated signaling pathway	4

Biological_process	odontogenesis	5
Biological_process	positive regulation of smooth muscle contraction	3
Biological_process	regulation of cell growth	8
Biological_process	organic acid transport	7
Biological_process	negative regulation of signal transduction in absence of ligand	3
Biological_process	negative regulation of extrinsic apoptotic signaling pathway in absence of ligand	3
Biological_process	neural crest cell development	4
Biological_process	axon extension involved in axon guidance	3
Biological_process	neuron projection extension involved in neuron projection guidance	3
Biological_process	mesenchymal cell development	4
Biological_process	stem cell development	4
Biological_process	organic cation transport	3
Biological_process	nerve development	4
Biological_process	neural crest cell differentiation	4
Biological_process	regulation of microtubule polymerization or depolymerization	4
Biological_process	regulation of cellular extravasation	3
Biological_process	appendage morphogenesis	5
Biological_process	limb morphogenesis	5
Biological_process	neurotransmitter loading into synaptic vesicle	2
Biological_process	negative regulation of mitochondrial outer membrane permeabilization involved in apoptotic signaling pathway	2
Biological_process	head morphogenesis	3
Biological_process	organic anion transport	7
Biological_process	extracellular matrix assembly	3

Biological_process	regulation of blood circulation	6
Biological_process	regulation of microtubule nucleation	2
Biological_process	limb bud formation	2
Biological_process	regulation of nucleotide-binding oligomerization domain containing signaling pathway	2
Biological_process	negative regulation of inclusion body assembly	2
Biological_process	transforming growth factor beta receptor signaling pathway	5
Biological_process	regulation of tube diameter	5
Biological_process	blood vessel diameter maintenance	5
Biological_process	regulation of tube size	5
Biological_process	branching morphogenesis of an epithelial tube	5
Biological_process	azole transmembrane transport	2
Biological_process	regulation of uterine smooth muscle contraction	2
Biological_process	positive regulation of tumor necrosis factor-mediated signaling pathway	2
Biological_process	regulation of heart rate	4
Biological_process	positive regulation of epithelial to mesenchymal transition	3
Biological_process	regulation of extrinsic apoptotic signaling pathway in absence of ligand	3
Biological_process	cytoplasmic pattern recognition receptor signaling pathway	3
Biological_process	positive regulation of muscle contraction	3
Biological_process	regulation of apoptotic signaling pathway	7
Biological_process	vasoconstriction	4
Biological_process	positive regulation of neuron differentiation	4
Biological_process	transforming growth factor beta1 production	2
Biological_process	skin morphogenesis	2

Biological_process	elastic fiber assembly	2
Biological_process	male mating behavior	2
Biological_process	bone trabecula formation	2
Biological_process	uterine smooth muscle contraction	2
Biological_process	cytokine-mediated signaling pathway	7
Biological_process	positive regulation of amine transport	3
Biological_process	connective tissue development	6
Biological_process	appendage development	5
Biological_process	limb development	5
Biological_process	positive regulation of organic acid transport	3
Biological_process	extracellular matrix organization	6
Biological_process	regulation of amine transport	4
Biological_process	extracellular structure organization	6
Biological_process	trabecula morphogenesis	3
Biological_process	mesenchyme morphogenesis	3
Biological_process	external encapsulating structure organization	6
Biological_process	regulation of cytokine-mediated signaling pathway	4
Biological_process	monocarboxylic acid transport	4
Biological_process	positive regulation of renal sodium excretion	2
Biological_process	regulation of ribonuclease activity	2
Biological_process	negative regulation of transcription from RNA polymerase II promoter in response to stress	2
Biological_process	bone mineralization	4
Biological_process	positive regulation of anion transport	3
Biological_process	microtubule polymerization or depolymerization	4
Biological_process	renal system process	4

Biological_process	body morphogenesis	3
Biological_process	cartilage development	5
Biological_process	positive regulation of prostaglandin secretion	2
Biological_process	regulation of microtubule polymerization	3
Biological_process	osteoblast differentiation	5
Biological_process	regulation of response to cytokine stimulus	4
Biological_process	forebrain generation of neurons	3
Biological_process	sodium ion homeostasis	3
Biological_process	drinking behavior	2
Biological_process	bone trabecula morphogenesis	2
Biological_process	regulation of ossification	4
Biological_process	morphogenesis of a branching epithelium	5
Biological_process	positive regulation of ion transport	6
Biological_process	acid secretion	3
Biological_process	ear morphogenesis	4
Biological_process	regulation of neuron differentiation	5
Biological_process	response to heat	4
Biological_process	regulation of prostaglandin secretion	2
Biological_process	positive regulation of catecholamine secretion	2
Biological_process	protein refolding	2
Biological_process	sodium-independent organic anion transport	2
Biological_process	cellular response to mineralocorticoid stimulus	2
Biological_process	regulation of inclusion body assembly	2
Biological_process	regulation of mitotic spindle assembly	2
Biological_process	visual perception	4
Biological_process	endochondral bone morphogenesis	3

Biological_process	reactive oxygen species metabolic process	5
Biological_process	stem cell differentiation	5
Biological_process	regulation of catecholamine secretion	3
Biological_process	nephron tubule formation	2
Biological_process	axon extension	4
Biological_process	sensory perception of light stimulus	4
Biological_process	morphogenesis of a branching structure	5
Biological_process	pattern specification process	7
Biological_process	catecholamine secretion	3
Biological_process	epithelial to mesenchymal transition	4
Biological_process	response to retinoic acid	4
Biological_process	positive regulation of icosanoid secretion	2
Biological_process	chaperone-mediated protein complex assembly	2
Biological_process	negative regulation of digestive system process	2
Biological_process	response to mineralocorticoid	3
Biological_process	amide transport	6
Biological_process	prostaglandin secretion	2
Biological_process	regulation of mitochondrial outer membrane permeabilization involved in apoptotic signaling pathway	2
Biological_process	negative regulation of endoplasmic reticulum stress-induced intrinsic apoptotic signaling pathway	2
Biological_process	positive regulation of leukocyte migration	4
Biological_process	positive regulation of secretion by cell	6
Biological_process	sensory perception of pain	4
Biological_process	negative regulation of transforming growth factor beta receptor signaling pathway	3

Biological_process	signal transduction in absence of ligand	3
Biological_process	cellular response to retinoic acid	3
Biological_process	extrinsic apoptotic signaling pathway in absence of ligand	3
Biological_process	positive regulation of cellular extravasation	2
Biological_process	cell migration in hindbrain	2
Biological_process	regulation of odontogenesis	2
Biological_process	cellular response to steroid hormone stimulus	5
Biological_process	monovalent inorganic cation homeostasis	4
Biological_process	regulation of microtubule cytoskeleton organization	4
Biological_process	regulation of postsynaptic membrane potential	4
Biological_process	regionalization	6
Biological_process	parturition	2
Biological_process	transmembrane receptor protein serine/threonine kinase signaling pathway	6
Biological_process	leukocyte migration	6
Cellular_component	fibrillar collagen trimer	5
Cellular_component	banded collagen fibril	5
Cellular_component	complex of collagen trimers	5
Cellular_component	collagen trimer	5
Cellular_component	extracellular matrix	9
Cellular_component	external encapsulating structure	9
Cellular_component	collagen-containing extracellular matrix	7
Cellular_component	clathrin-coated endocytic vesicle	3
Cellular_component	secretory granule	8
Cellular_component	membrane raft	8

Cellular_component	membrane microdomain	8
Cellular_component	clathrin-coated vesicle	4
Cellular_component	basal part of cell	6
Cellular_component	acetylcholine-gated channel complex	2
Cellular_component	cholinergic synapse	2
Cellular_component	synaptic vesicle	5
Cellular_component	plasma membrane signaling receptor complex	4
Cellular_component	receptor complex	6
Molecular_function	platelet-derived growth factor binding	4
Molecular_function	extracellular matrix structural constituent	5
Molecular_function	growth factor binding	6
Molecular_function	G protein-coupled receptor binding	8
Molecular_function	receptor ligand activity	9
Molecular_function	signaling receptor activator activity	9
Molecular_function	signaling receptor regulator activity	9
Molecular_function	organic cation transmembrane transporter activity	3
Molecular_function	protease binding	5
Molecular_function	azole transmembrane transporter activity	2
Molecular_function	cell adhesion molecule binding	6
Molecular_function	cytokine activity	5
Molecular_function	acetylcholine binding	2
Molecular_function	anion transmembrane transporter activity	6
Molecular_function	transmitter-gated ion channel activity involved in regulation of postsynaptic membrane potential	3
Molecular_function	neurotransmitter receptor activity involved in regulation of postsynaptic membrane potential	3
Molecular_function	transmitter-gated ion channel activity	3

Molecular_function	transmitter-gated channel activity	3
Molecular_function	hormone activity	4
Molecular_function	DNA-binding transcription factor binding	7
Molecular_function	acetylcholine-gated cation-selective channel activity	2
Molecular_function	RNA polymerase II-specific DNA-binding transcription factor binding	6
Molecular_function	sodium-independent organic anion transmembrane transporter activity	2
Molecular_function	acetylcholine receptor activity	2
Molecular_function	extracellular ligand-gated ion channel activity	3
Molecular_function	postsynaptic neurotransmitter receptor activity	3
Molecular_function	neurotransmitter binding	2
Molecular_function	vitamin transmembrane transporter activity	2
Molecular_function	misfolded protein binding	2
Molecular_function	organic anion transmembrane transporter activity	4
