# Supplementary material for: Transcriptomic analysis of the cerebral hippocampal tissue in spontaneously hypertensive rats exposed to acute hypobaric hypoxia: associations with inflammation and energy metabolism
Source: Sci Rep. 2023 Mar 6;13:3681. doi: 10.1038/s41598-023-30682-0 (PMC9988845; doi:10.1038/s41598-023-30682-0)
Supplement: Supplementary file 8 — Supplementary Information 8. [file 41598_2023_30682_MOESM8_ESM.pdf]

Table S6. Summary of Clusters of orthologous groups of proteins (COG) classification analysis of 112 differential expression genes (DEGs).

Type	Functional Categories	Frequency
METABOLISM	[C]Energy production and conversion	6
METABOLISM	[E]Amino acid transport and metabolism	7
METABOLISM	[F]Nucleotide transport and metabolism	3
METABOLISM	[G]Carbohydrate transport and metabolism	16
METABOLISM	[H]Coenzyme transport and metabolism	2
METABOLISM	[I]Lipid transport and metabolism	4
INFORMATION STORAGE AND PROCESSING	[J]Translation, ribosomal structure and biogenesis	32
INFORMATION STORAGE AND PROCESSING	[K]Transcription	5
INFORMATION STORAGE AND PROCESSING	[L]Replication, recombination and repair	22
CELLULAR PROCESSES AND SIGNALING	[M]Cell wall/membrane/envelope biogenesis	10
CELLULAR PROCESSES AND SIGNALING	[O]Posttranslational modification, protein turnover, chaperones	14
METABOLISM	[P]Inorganic ion transport and metabolism	3
METABOLISM	[Q]Secondary metabolites biosynthesis, transport and catabolism	1
POORLY CHARACTERIZED	[R]General function prediction only	94
POORLY CHARACTERIZED	[S]Function unknown	8
CELLULAR PROCESSES AND SIGNALING	[T]Signal transduction mechanisms	8
CELLULAR PROCESSES AND SIGNALING	[U]Intracellular trafficking, secretion, and vesicular transport	4
CELLULAR PROCESSES AND SIGNALING	[V]Defense mechanisms	5
