# Supplementary material for: Transcriptomic analysis of the cerebral hippocampal tissue in spontaneously hypertensive rats exposed to acute hypobaric hypoxia: associations with inflammation and energy metabolism
Source: Sci Rep. 2023 Mar 6;13:3681. doi: 10.1038/s41598-023-30682-0 (PMC9988845; doi:10.1038/s41598-023-30682-0)
Supplement: Supplementary file 9 — Supplementary Information 9. [file 41598_2023_30682_MOESM9_ESM.pdf]

Table S7. Summary of Kyoto Encyclopedia of Genes and Genomes (KEGG) pathways analysis of 112 differential expression genes (DEGs).

Pathway ID	Description	Number of DEGs in category
rno04926	Relaxin signaling pathway	8
rno04151	PI3K-Akt signaling pathway	10
rno05146	Amoebiasis	5
rno04933	AGE-RAGE signaling pathway in diabetic complications	5
rno04080	Neuroactive ligand-receptor interaction	9
rno04510	Focal adhesion	6
rno05205	Proteoglycans in cancer	6
rno04512	ECM-receptor interaction	4
rno04014	Ras signaling pathway	6
rno04064	NF-kappa B signaling pathway	4
rno04916	Melanogenesis	4
rno04974	Protein digestion and absorption	4
rno04659	Th17 cell differentiation	4
rno05134	Legionellosis	3
rno05165	Human papillomavirus infection	7
rno05217	Basal cell carcinoma	3
rno04010	MAPK signaling pathway	6
rno05162	Measles	4
rno05224	Breast cancer	4
rno05226	Gastric cancer	4
rno04061	Viral protein interaction with cytokine and cytokine receptor	3
rno04150	mTOR signaling pathway	4
rno04658	Th1 and Th2 cell differentiation	3
rno04310	Wnt signaling pathway	4
rno05340	Primary immunodeficiency	2
rno04650	Natural killer cell mediated cytotoxicity	3
rno05225	Hepatocellular carcinoma	4
rno04060	Cytokine-cytokine receptor interaction	5
rno04216	Ferroptosis	2
rno05145	Toxoplasmosis	3
rno04725	Cholinergic synapse	3
rno04066	HIF-1 signaling pathway	3
rno04722	Neurotrophin signaling pathway	3
rno04380	Osteoclast differentiation	3
rno05417	Lipid and atherosclerosis	4
rno05415	Diabetic cardiomyopathy	4
rno04611	Platelet activation	3
rno05167	Kaposi sarcoma-associated herpesvirus infection	4

rno04915	Estrogen signaling pathway	3
rno04550	Signaling pathways regulating pluripotency of stem cells	3
rno04213	Longevity regulating pathway - multiple species	2
rno05163	Human cytomegalovirus infection	4
rno05418	Fluid shear stress and atherosclerosis	3
rno04520	Adherens junction	2
rno04390	Hippo signaling pathway	3
rno04934	Cushing syndrome	3
rno05412	Arrhythmogenic right ventricular cardiomyopathy	2
rno00533	Glycosaminoglycan biosynthesis - keratan sulfate	1
rno05220	Chronic myeloid leukemia	2
rno01521	EGFR tyrosine kinase inhibitor resistance	2
rno05323	Rheumatoid arthritis	2
rno04612	Antigen processing and presentation	2
rno04062	Chemokine signaling pathway	3
rno05235	PD-L1 expression and PD-1 checkpoint pathway in cancer	2
rno05206	MicroRNAs in cancer	4
rno00360	Phenylalanine metabolism	1
rno04640	Hematopoietic cell lineage	2
rno04727	GABAergic synapse	2
rno05410	Hypertrophic cardiomyopathy	2
rno04657	IL-17 signaling pathway	2
rno04976	Bile secretion	2
rno05215	Prostate cancer	2
rno04660	T cell receptor signaling pathway	2
rno04015	Rap1 signaling pathway	3
rno04977	Vitamin digestion and absorption	1
rno04024	cAMP signaling pathway	3
rno04668	TNF signaling pathway	2
rno04966	Collecting duct acid secretion	1
rno01523	Antifolate resistance	1
rno04726	Serotonergic synapse	2
rno04215	Apoptosis - multiple species	1
rno05166	Human T-cell leukemia virus 1 infection	3
rno03040	Spliceosome	2
rno04068	FoxO signaling pathway	2
rno04936	Alcoholic liver disease	2
rno05135	Yersinia infection	2
rno04210	Apoptosis	2
rno05132	Salmonella infection	3
rno00350	Tyrosine metabolism	1
rno04960	Aldosterone-regulated sodium reabsorption	1
rno05216	Thyroid cancer	1
rno04270	Vascular smooth muscle contraction	2

rno00591	Linoleic acid metabolism	1
rno05033	Nicotine addiction	1
rno05143	African trypanosomiasis	1
rno05219	Bladder cancer	1
rno04672	Intestinal immune network for IgA production	1
rno04144	Endocytosis	3
rno05030	Cocaine addiction	1
rno00380	Tryptophan metabolism	1
rno04217	Necroptosis	2
rno04141	Protein processing in endoplasmic reticulum	2
rno05164	Influenza A	2
rno00600	Sphingolipid metabolism	1
rno02010	ABC transporters	1
rno04623	Cytosolic DNA-sensing pathway	1
rno05144	Malaria	1
rno05034	Alcoholism	2
rno04978	Mineral absorption	1
rno04370	VEGF signaling pathway	1
rno05213	Endometrial cancer	1
rno04360	Axon guidance	2
rno04622	RIG-I-like receptor signaling pathway	1
rno05321	Inflammatory bowel disease	1
rno05031	Amphetamine addiction	1
rno05221	Acute myeloid leukemia	1
rno05211	Renal cell carcinoma	1
rno05140	Leishmaniasis	1
rno04920	Adipocytokine signaling pathway	1
rno04924	Renin secretion	1
rno05214	Glioma	1
rno04917	Prolactin signaling pathway	1
rno05212	Pancreatic cancer	1
rno04662	B cell receptor signaling pathway	1
rno05100	Bacterial invasion of epithelial cells	1
rno04721	Synaptic vesicle cycle	1
rno00590	Arachidonic acid metabolism	1
rno04810	Regulation of actin cytoskeleton	2
rno04012	ErbB signaling pathway	1
rno05207	Chemical carcinogenesis - receptor activation	2
rno05208	Chemical carcinogenesis - reactive oxygen species	2
rno05210	Colorectal cancer	1
rno05032	Morphine addiction	1
rno05222	Small cell lung cancer	1
rno04620	Toll-like receptor signaling pathway	1
rno01522	Endocrine resistance	1

rno05414	Dilated cardiomyopathy	1
rno00564	Glycerophospholipid metabolism	1
rno05231	Choline metabolism in cancer	1
rno05142	Chagas disease	1
rno04931	Insulin resistance	1
