# Supplementary material for: Transcriptomic analysis of the cerebral hippocampal tissue in spontaneously hypertensive rats exposed to acute hypobaric hypoxia: associations with inflammation and energy metabolism
Source: Sci Rep. 2023 Mar 6;13:3681. doi: 10.1038/s41598-023-30682-0 (PMC9988845; doi:10.1038/s41598-023-30682-0)
Supplement: Supplementary file 10 — Supplementary Information 10. [file 41598_2023_30682_MOESM10_ESM.pdf]

Table S8. Summary of Kyoto Encyclopedia of Genes and Genomes (KEGG) pathways analysis of up-regulated differential expression genes (DEGs).

Pathway ID	Description	Number of DEGs in category
rno04014	Ras signaling pathway	5
rno05340	Primary immunodeficiency	2
rno01521	EGFR tyrosine kinase inhibitor resistance	2
rno04658	Th1 and Th2 cell differentiation	2
rno04650	Natural killer cell mediated cytotoxicity	2
rno04151	PI3K-Akt signaling pathway	3
rno04659	Th17 cell differentiation	2
rno04066	HIF-1 signaling pathway	2
rno04926	Relaxin signaling pathway	2
rno04510	Focal adhesion	2
rno04015	Rap1 signaling pathway	2
rno05167	Kaposi sarcoma-associated herpesvirus infection	2
rno04966	Collecting duct acid secretion	1
rno05206	MicroRNAs in cancer	2
rno04010	MAPK signaling pathway	2
rno00591	Linoleic acid metabolism	1
rno05143	African trypanosomiasis	1
rno05219	Bladder cancer	1
rno04216	Ferroptosis	1
