# Supplementary material for: Transcriptomic analysis of the cerebral hippocampal tissue in spontaneously hypertensive rats exposed to acute hypobaric hypoxia: associations with inflammation and energy metabolism
Source: Sci Rep. 2023 Mar 6;13:3681. doi: 10.1038/s41598-023-30682-0 (PMC9988845; doi:10.1038/s41598-023-30682-0)
Supplement: Supplementary file 11 — Supplementary Information 11. [file 41598_2023_30682_MOESM11_ESM.pdf]

Table S9. Summary of Kyoto Encyclopedia of Genes and Genomes (KEGG) pathways analysis of down-regulated differential expression genes (DEGs).

Pathway ID	Description	Number of DEGs in category
rno04926	Relaxin signaling pathway	6
rno04080	Neuroactive ligand-receptor interaction	9
rno05146	Amoebiasis	5
rno04512	ECM-receptor interaction	4
rno04916	Melanogenesis	4
rno04151	PI3K-Akt signaling pathway	7
rno04933	AGE-RAGE signaling pathway in diabetic complications	4
rno04974	Protein digestion and absorption	4
rno05134	Legionellosis	3
rno05205	Proteoglycans in cancer	5
rno05217	Basal cell carcinoma	3
rno04150	mTOR signaling pathway	4
rno05165	Human papillomavirus infection	6
rno04310	Wnt signaling pathway	4
rno04064	NF-kappa B signaling pathway	3
rno04510	Focal adhesion	4
rno05145	Toxoplasmosis	3
rno04725	Cholinergic synapse	3
rno05417	Lipid and atherosclerosis	4
rno05415	Diabetic cardiomyopathy	4
rno04380	Osteoclast differentiation	3
rno04611	Platelet activation	3
rno04550	Signaling pathways regulating pluripotency of stem cells	3
rno05162	Measles	3
rno05224	Breast cancer	3
rno05226	Gastric cancer	3
rno04213	Longevity regulating pathway - multiple species	2
rno04390	Hippo signaling pathway	3
rno04934	Cushing syndrome	3
rno04060	Cytokine-cytokine receptor interaction	4
